# Possible Biomarkers in Blood for Crohn's Disease: Oxidative Stress and MicroRNAs—Current Evidences and Further Aspects to Unravel

**DOI:** 10.1155/2016/2325162

**Published:** 2015-12-28

**Authors:** Inés Moret-Tatay, Marisa Iborra, Elena Cerrillo, Luis Tortosa, Pilar Nos, Belén Beltrán

**Affiliations:** ^1^Inflammatory Bowel Disease Research Group, IIS Hospital La Fe, Avenida Fernado Abril Martorell, No. 106, 46026 Valencia, Spain; ^2^CIBERehd, Centro de Investigación Biomédica en Red de Enfermedades Hepáticas y Digestivas, Barcelona, Spain; ^3^Gastroenterology Department, La Fe University and Polytechnic Hospital, Avenida Fernado Abril Martorell, No. 106, 46026 Valencia, Spain

## Abstract

Crohn's disease (CD) is an inflammatory disorder characterised by a transmural inflammation of the intestinal wall. Although the physiopathology of the disease is not yet fully understood, it is clear that the immune response plays an important role in it. This hyperreactive immune system is accompanied by the presence of unregulated reactive oxygen species (ROS). These elements are modulated in normal conditions by different elements, including enzymes that function as antioxidant defences preventing the harmful effects of ROS. However, in CD there is an imbalance between ROS production and these antioxidant elements, resulting in oxidative stress (OxS) phenomena. In fact, now OxS is being considered more a potential etiological factor for Crohn's disease rather than a concomitant effect in the disease. The persistence of the OxS can also be influencing the evolution of the disease. Furthermore, the epigenetic mechanisms, above all microRNAs, are being considered key elements in the pathogenesis of CD. These elements and the presence of OxS have also been linked to several diseases. We, therefore, describe in this review the most significant findings related to oxidative stress and microRNAs profiles in the peripheral blood of CD patients.

## 1. Introduction

Crohn's disease (CD) is, together with ulcerative colitis (UC), one of the two major forms of inflammatory bowel disease (IBD). They are both conditions characterized by chronic inflammation of the digestive tract. Although they share many similarities, there are key differences between the two diseases. While UC is limited to the colon, the inflammation is continuous and only affects the colonic mucosa, and CD is characterised by transmural and discontinuous inflammation of the gastrointestinal tract, which most commonly affects the terminal ileum and proximal colon [1, 2]. Although its precise etiology remains unclear, it is thought that interactions among genetic factors, the host immune system and environmental/microbiota agents, play crucial roles in disturbing the intestinal homeostasis, leading to the dysregulated inflammatory responses of the gut. Furthermore, this hyperreactive immune system is accompanied by abnormally high levels of reactive oxygen species (ROS), and the resulting oxidative stress (OxS) phenomenon has been considered as a potential etiological factor for CD.

ROS are natural products formed during the oxygen metabolism and generation of H_2_O. The main prooxidant agents are ROS formed by unstable forms of oxygen: superoxide (O_2_^−^), hydrogen peroxide (H_2_O_2_), and hydroxyl radicals (OH^•^). These molecules (also known as free radicals) are a highly reactive species due to having unpaired electrons in the outermost orbital electron shell, especially OH^•^, the most important ROS involved in cellular oxidative damage. In addition, cells can tolerate moderate oxidative loads by increasing gene expression to upregulate their reductive defence systems and restore the oxidant/antioxidant balance. But when this increased synthesis cannot be achieved due to damage to enzymes, or substrate limitations, or when the increased or prolonged oxidative load is overwhelming, an imbalance persists and the result is oxidative stress [3–6].

In such situations, ROS can damage various cellular components, being the membrane lipids, proteins, and nucleic acids the most susceptible to oxidation, and therefore the prime targets for ROS. This will result in harmful effects on cellular activity at different levels, which ultimately will affect the homeostasis and cellular metabolism, and even may lead to the death of the affected cells. In this sense, OxS induces lipid peroxidation through their action of free radicals and peroxides with polyunsaturated fatty acids (PUFAs) of the cellular membranes, resulting in new products formation, such as malondialdehyde (MDA), which can cause protein damage by reactions with lysine amino groups, histidine imidazole groups, or cysteine sulfhydryl groups [7]. Therefore, the end products of lipid peroxidation can affect membrane proteins by cross-linkage, rendering them useless as receptors or enzymes. In a similar manner, ROS can cause oxidative modifications in nuclear and mitochondrial DNA bases. The major sign of these specific lesions caused by ROS is the formation (and identification in DNA) of oxidatively modified bases, such as 8-hydroxy-2′-deoxyguanosine (8-OHdG), which is one of the predominant forms of free radical-induced oxidative lesions and has therefore been widely used as a biomarker for OxS and carcinogenesis [8, 9].

So, interestingly, the breakdown products of these oxidation processes may be employed as useful biomarkers for identifying the effect of endogenous OxS. These biomarkers have been reported to be present in Crohn's disease patients [10]: an increase of lipid peroxidation (MDA detection) and damage to the DNA (8-OHdG), as will be explained in more detail below.

On the other hand, the presence of OxS has also been linked with epigenetic mechanisms in several diseases, above all microRNAs (miRNAs), and there is an increasing interest in exploring their joint contribution to the pathogenesis of CD. miRNAs are short strands of noncoding RNA that posttranscriptionally regulate gene expression and are being considered key elements in the pathogenesis of CD [11–13]. It is estimated that miRNAs regulate more than 60% of protein coding mRNAs, identifying also that circulating miRNAs can be useful noninvasive biomarkers in several diseases including IBD [14–17].

There are current studies indicating that the miRNAs expression can be sensitive to the presence of intracellular H_2_O_2_ levels. Epigenetic regulation at the DNA level is an important mechanism involved in H_2_O_2_-mediated expression changes of multiple genes, indicating that miRNA expression is very sensitive to H_2_O_2_ stimulation [18]. For example, in smooth muscle cells, the cellular treatment with hydrogen peroxide resulted in an upregulation of microRNA-21 [18]. In addition, the expression of miR-181a in H_2_O_2_-treated H9c2 cells (cell line derived from rat heart tissue) was markedly upregulated [19]. In that context, miRNAs could be modulating intracellular pathways formed by the participation of multiple proteins. That would be the case of ROS-mediated events [19]. Furthermore, the relationship of mitochondrial dysfunction, defined as the result of the increased production of ROS in mitochondria, the accumulation of mitochondrial DNA damage, and the progressive respiratory chain alteration, plus the altered expression of miRNA is now being established for the onset of some diseases [20]. What happens in the case of CD needs to be deeply studied.

Unraveling the signaling events initiated at the cellular level by oxidative free radicals, as well as the changes that occur in microRNA expression, is important not only because of the need to better understand the disease pathogenesis, but also because of its implications in the search for new biomarkers and the design of new therapeutic targets. We, therefore, review in the following sections the most current and significant findings related to oxidative stress and microRNA profiles in peripheral blood, with the main focus on the CD patients.

## 2. Oxidative Stress Biomarkers in Blood of Crohn's Disease Patients

Among the different pathogenic elements in CD, the dysfunctional immune regulation and the presence of OxS emerge to be key elements highly implied in the disease. Currently, the evidence confirms that the presence of OxS in CD is not limited to the intestinal mucosa and therefore the accumulation of ROS and/or their oxidation products are also present in the peripheral blood [21–26]. Initially, as inflammation involves the formation of ROS, they were proposed to be elements underlying the disease. This thought is now being changed, after observing in experimental protocols (employing animal models and humans samples) that ROS and OxS play a critical role in the early stages and in the progression of the disease [5].

Recently, it has been observed that ROS are directly involved in the tissue injury, especially when the antioxidant defences are overwhelmed. During the inflammation process, the presence of high levels of ROS can saturate the antioxidant defense systems [27]. This fact has been described in the intestinal mucosa, where the relatively small amounts of antioxidant enzymes are overwhelmed during the active inflammation [28], confirming an imbalanced and inefficient endogenous antioxidant response to ROS in IBD patients [29]. As these processes can play an important pathogenic effect in IBD, new research areas are focused in their study to unravel the pathogenic origin of IBD [4, 30]. The signalling events initiated at the cellular level by oxidative free radicals, as well as the physiological responses to such stress, are important factors to be considered for better understanding the disease and for discovery of novel disease biomarkers. In this context, two characteristic effects are commonly present in all CD patients: the decreased antioxidant status and the elevated markers of OxS [3, 22]. Due to their importance, an emerging interest in their study has been observed.

### 2.1. Decreased Antioxidant Status in Crohn's Disease

**Figure 1:**
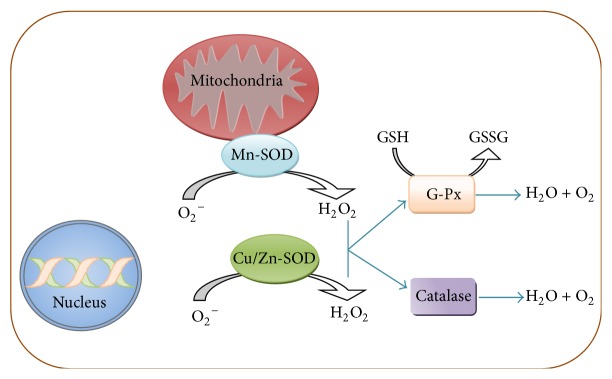
Main antioxidant enzymes and their substrates. These enzymes have altered functions in Crohn's disease.

Antioxidants are defined as substances that can delay or prevent oxidative damage caused by the presence of ROS [31]. In blood, antioxidants can be classified into plasma (extracellular antioxidants) and intracellular antioxidants [3, 5]. Plasma antioxidants include vitamins (A, C, and E), serum bilirubin, ceruloplasmin, and uric acid, which help to protect cells they are in contact with and components of the plasma [32]. The intracellular antioxidants are mainly glutathione (GSH) and the antioxidant enzymes, superoxide dismutase (SOD), catalase (CAT), and glutathione peroxidase (G-Px), which are essentials for the survival of the organisms and for their health. That means that antioxidants can be nonenzymatic, such as vitamins (A, C, and E), minerals (selenium and zinc), and other compounds (GSH, uric acid, ubiquinol, serum bilirubin, and so on), and enzymatic (Figure 1): SOD, CAT, and G-Px as the most important [33]. Some of these elements are endogenous antioxidants, such as serum bilirubin, glutathione, G-Px, cytosolic (Cu/Zn-SOD) and mitochondrial (Mn-SOD) SOD, and CAT, whereas others are exogenous antioxidants, such as vitamins, carotenoids, and polyphenols. Both endogenous and exogenous antioxidants work together to maintain redox homeostasis [31].

From a mechanistic point of view, the antioxidants can be classified as preventing antioxidants, scavenging antioxidants, and repair/de novo antioxidants [4]. The two main functions of antioxidant enzymes would be suppressing the formation of reactive species (CAT), or removing active species before they attack biological elements (SOD). Below, these nonenzymatic and enzymatic antioxidant elements in the context of Crohn's disease are discussed.

The intestine has low stores of endogenous antioxidants to help against free radicals [24]. That means that any imbalance within these elements, by decreasing the antioxidant levels and/or by increasing the ROS levels, can subsequently result in oxidative stress in the gut [33]. Interestingly, antioxidant performance analyzed in peripheral blood reflects oxidative stress in the target tissue and therefore facilitates identifying the presence of OxS in CD. Some authors have reported that CD patients have decreased blood levels of the antioxidant vitamins [34]. However, up to now it is not clear if it is a consequence of an impaired nutritional status (malnutrition) or it can be an independent factor [35]. In agreement with this idea, low levels of vitamins A and E have been found in CD patients with low, normal, and overweight [24]. Similar results have been also obtained when circulating vitamin D is measured in these patients [36]. In this case, the relationship between low levels of vitamin D in CD and malabsorption, from short gut syndrome in operated patients or for gut inflammation, has been established [37]. Also, other factors such as decreased dietary intake and limited exposure to sunlight seem to be related to hypovitaminosis D [38]. Interestingly, another important antioxidant element, selenium, is also commonly decreased in CD patients [39]. Some authors have hypothesized, based on animal studies, that the lower selenium content in plasma is presumably caused by decreased absorption in Crohn's disease patients [39].

The detailed study of peripheral immune cells, particularly neutrophils, which are within the first immune cells migrating to the site of infection, demonstrated that there is a significant change in the metabolism of glutathione (GSH) in the disease [40]. Other elements such as serum bilirubin, an important endogenous antioxidant, are also decreased presumably as a result of the increased oxidative stress [41]. Strikingly, some cytokines, small proteins that allow communication between different immune cells, have been demonstrated to be implied in the oxidative stress phenomena. In fact, various cytokines plus products of protein damage due to oxidative stress (3-chlorotyrosine and 3-nitrotyrosine) have characteristic profiles in serum of CD patients [42]. Between all them, the proinflammatory cytokine TNF*α* (tumor necrosis factor alpha), which plays an important role in the disease, has been clearly linked to the OxS. Its blockage by anti-TNF therapies results in decreasing levels of lipid peroxidation (conjugated dienes) and increasing levels of the oxygen species scavenger ceruloplasmin [43].

The levels of extracellular antioxidants can be low in the blood of CD patients, but also the intracellular antioxidants are decreased in the peripheral blood mononuclear cells (PBMC) of CD patients [21, 44]. As mentioned before, the main antioxidant enzymes are G-Px, SOD, and CAT (Figure 1). Referring to G-Px, it was observed that its activity is decreased in the disease, which also can correspond with the disease activity and inflammation [44]. Furthermore, the impaired G-Px is accompanied by an increase in the transcription mediated by nuclear factor-*κ*B (NF-*κ*B) and increased production of proinflammatory cytokines [45]. Also important to consider are the relationship of G-PX with selenium and the fact that this element is decreased in CD [39]. The selenium deficiency may interfere with G-Px antioxidant capacity, as this enzyme requires selenium as a cofactor for activity [46]. Also, biosynthesis of G-Px can be affected by selenium depletion [47]. However, the results of G-Px have been demonstrated to be diverse when analysing plasma and serum samples of CD patients. For example, serum G-Px enzyme activity is higher in CD patients than in healthy controls, differences that disappeared when patients were in the inactive phase (with bowel rest and no signs of symptoms of the disease) [48]. Nevertheless, in a more recent study no differences of plasma G-Px were found between CD patients with active (clinical activity, sign, and symptoms of the disease) or inactive phase and healthy controls [49]. Meanwhile, another study found decreased G-Px activities in patients with CD [44]. All this diverse information indicates that it is essential to consider different key factors affecting the results: the type of the analysed sample, the time point when it is collected, and the protocol employed to process it. All these factors can influence the result, and, therefore, these aspects have to be taken into account before interpreting the enzymatic activities.

There are three forms of SOD in humans: cytosolic (Cu/Zn-SOD), mitochondrial (Mn-SOD), and extracellular (EC-SOD). Most studies indicate that SOD enzyme remains unchanged in plasma and serum samples of IBD patients or even increases its activity [50]. Other studies have observed [44] that Cu/Zn-SOD (SOD1) also correlates indirectly with CD activity and erythrocyte sedimentation rate (ESR). In that context, our group has also addressed studies to analyse this enzyme in samples of PBMC from CD patients at different stages of the disease, and in healthy controls. Our most important findings are that CD patients at onset have higher SOD activity than healthy controls, but it returns to normal levels when patients are in remission or in inactivity [23]. However, other studies have reported that lower levels of Cu/Zn-SOD protein and activity are present in peripheral blood granulocytes of IBD patients [51]. Again, factors such as if patients included in the studies were at the onset of the disease and naïve to specific medications, or they were in the active/or inactive phase and under treatment, can determine the differences between all these studies.

It is noteworthy that the G-Px and SOD enzymes have been widely studied for IBD, but not so for the other antioxidant enzyme: catalase (Figure 1). The few studies reported so far indicate that this enzyme activity is decreased in plasma and serum of IBD patients [50]. With the aim of better understanding the role of CAT in the disease, we have characterised this enzyme. We observed that under active disease, peripheral blood lymphocytes (PBL) exhibited a significant increase in the catalase substrate: H_2_O_2_ [22, 23]. A deeper analysis of this result gave rise to the observation of a permanent enzymatic activity inhibition, which was independent of the disease stage (active or inactive). This lower catalase activity was related to a lower gene expression in CD than in healthy controls, which in turn resulted in a persistent oxidative stress [23] as H_2_O_2_ levels were not completely removed (despite the increased G-Px activity). In any case, the role of CAT enzyme must be more complex than just helping with H_2_O_2_ detoxifying, and its implication in other cellular pathways, as apoptosis, is currently being under study. In addition, it is known that CAT may be exerting a role in the pathophysiology of CD, as CAT is one of the cytoplasmic antigens of the antineutrophil cytoplasmic antibodies (ANCAs). These antibodies have been associated with several CD clinical phenotypes [52].

Other determinant aspects to consider when analysing antioxidant enzymes, as pointed out before, are that SOD, G-Px, and CAT are predominantly in the intracellular space (Figure 1). In fact, human plasma has very little or no CAT [3] but can be released into the blood stream as part of the inflammatory response [53]. However, some isoforms of the G-Px (GPx3) and SOD (EC-SOD) may be present in human plasma [54, 55]. All of this means that measuring the activity of these enzymes directly in samples of plasma/serum, except for the extracellular isoforms, cannot be representative of the intracellular activity [44]. Therefore, peripheral blood cells used to determine the activity of these enzymes in CD seem to be the most appropriate specimen.

It has also been demonstrated that polymorphisms in the antioxidant enzymes (SOD and CAT) can lead to their lack of activity when measuring their activities in peripheral blood samples. In addition, some of these polymorphisms, rs1001179 and rs475043 (CAT), can be correlated with some clinical and demographic characteristics of the IBD patients [56]. Nevertheless, their implications in the pathogenesis of CD still need to be clarified.

### 2.2. Elevated Markers Related to Oxidative Stress in Crohn's Disease

ROS, which have always been believed to be harmful for the body, do in fact play a beneficial role and are useful species for immune cells to fight against pathogens located in the gut [5]. There is also enough scientific evidence showing that before reaching deleterious effects, ROS can exert a signalling function inside the cells regulating growth, differentiation, cell death, and inflammatory processes [57]. It has been observed that inhibition of ROS in dendritic cells produced a decrease of differentiation, indicating that ROS play a crucial role in this process [58]. In fact, the usual reaction to mild oxidative stress is to produce antioxidants to neutralize it. However, persistent high levels of ROS overwhelm the cellular mechanisms to avoid OxS damage, as previously mentioned.

Elevated plasma biomarkers of OxS include lipid peroxidation products [59] and oxidative damaged DNA in peripheral leukocytes. OxS induces lipid peroxidation through the action of free radicals and peroxides with polyunsaturated fatty acid (PUFAs) of the cellular membranes, resulting in malondialdehyde formation. These biomarkers of OxS have been reported to be present in Crohn's disease patients [10]: an increase of lipid peroxidation (MDA detection) and damage to the DNA (8-hydroxy-2′-deoxyguanosine, 8-OHdG). In CD, lipid peroxidation was found to correlate positively with SOD1 and interleukin-6 (IL-6) production but was negatively correlated with catalase [60]. The importance of increased levels of lipid peroxides has also been pointed out, which are secreted into the blood circulation from the gut of CD patients to produce systemic effects [3]. Furthermore, MDA was significantly increased during both active and inactive phases, though the second group tended to harbour lower levels.

Reactive oxygen and nitrogen species induce specific base modification, such as 8-oxo-dG and 8-nitro-dG, due to their high reactivity with nucleophilic sites on nucleobases [61]. The measurement of 8-OHdG incorporation into the DNA was showed to be permanently elevated in CD patients, though this increase was independent of the activity of the disease [23]. Similar results have been obtained [24] and elevated concentrations of 8-OHdG in blood leucocytes were not influenced by the disease activity, extension, or duration. On the other hand, it is also very useful to detect the protein damage through the carbonyl groups [33]. Levels of advanced oxidation protein products (AOPPs), formed by the action of chlorinated compounds, are also good indicators of disease activity, inflammatory and antioxidant response [62, 63].

Another reactive oxygen species to be considered is H_2_O_2_. This element, generated via induction of SOD, is directly related to catalase enzyme. This is an element that has been implied both in the cellular resistance to the cytotoxic effect of TNF-*α* and in the apoptotic process [64] at sublethal concentrations. Interestingly, in Crohn's disease, where TNF-*α* is well known to play a key role and where apoptosis of immune cells is impaired, the levels of H_2_O_2_ were elevated in PBMC during the active phase [23]. Also, increased levels of H_2_O_2_ in peripheral blood lymphocytes and monocytes significantly correlated with some inflammatory markers such as C-reactive protein (CRP) and fibrinogen in CD, indicating that inflammation is more pronounced when H_2_O_2_ concentration increases in those cells [23]. However, low doses of hydrogen peroxide can reduce the interferon-*γ* (IF-*γ*) production, a cytokine whose aberrant expression is associated with inflammatory diseases, in activated lymphocytes [65]. Thus, the exact role that this element plays in CD still needs to be clarified. On the other hand, the levels of other important ROS, such as nitric oxide (NO) or O_2_^−^, did not differ between CD and control subjects [22]. Again more studies are needed to confirm these results.

## 3. MicroRNAs Biomarkers in Blood of Crohn's Disease Patients

Recent investigations have attempted to clarify the involvement of epigenetic mechanisms such as DNA methylation, histone acetylation, and miRNAs in the pathogenesis of CD. MicroRNAs (miRNAs) are a class of small noncoding RNAs which regulate gene expression at posttranscriptional level. MiRNAs bind to complementary sequences in the 3′ untranslated region (UTR) of specific target mRNAs and can prevent protein synthesis [11]. In 1993, Lee et al. reported the first description of an miRNA, lin-4, in* Caenorhabditis elegans* [12]. Following the discovery of miRNAs, the number of publications regarding the biogenesis and functions of miRNAs has increased exponentially. To date, the last miRBase version (21 June 2014) of the miRNA sequence data base (http://www.mirbase.org/) includes over 28645 predicted miRNAs in species of plants, animals, and viruses [13].

miRNAs have been found in tissues, serum, plasma, and other body fluids (i.e., urine, tears, and ascetic and amniotic fluid), in a stable form that is protected from endogenous RNase activity due to their incorporation into the RNA-induced silencing complex (RISC) which is either free in blood or in exosomes [66]. For this reason, miRNA is resistant to harsh conditions and it is now being used as a biomarker for different pathologies (i.e., cancer, autoimmune disease, and inflammation) such as CD. Although it has been shown that miRNA levels are upregulated in both serum and cell lines, the levels of miRNA detected in serum and cell lines are different. Initial studies performed in cancer have identified that miRNA expression patterns seen in serum were not identical to those seen from miRNA taken directly from tissues. This result points towards a possible mechanism of tissue miRNA release into the circulation [66]. Several studies have identified a concordance between levels of both serum and plasma miRNA. This would greatly facilitate the clinical use to detect miRNA directly in serum and will be suitable for the investigation of miRNAs as blood-based biomarkers [67].

Although the majority of studies are focused on the potential role of miRNAs in the development of cancer, current studies have revealed that few miRNAs may be involved in the development and function of the immune system [14, 67–74]. These molecules are involved in the regulation of many biological processes, as well as in the induction of several cancers and chronic inflammatory disease [75, 76].

MiRNA-mediated gene regulation is implicated in normal cellular processes such as the cell cycle, differentiation, proliferation, apoptosis, and immune functions. It has already been shown that changes in miRNA expression can also regulate inflammatory responses in humans. Current studies explain that miRNA overexpression and/or inhibition can regulate the release of several proinflammatory chemokines. Specific miRNAs such as miR-132, miR-146, and miR-155 can be regulated by inflammatory mediators (NF-*κβ*, TNF-*α*, and IFN-*β*), and microbial components (lipopolysaccharides and flagellin) and a variety of Toll-like receptors ligands (TLR) led to physiological granulocyte/monocyte diffusion and growth during inflammation [71, 77].

Although the first studies were focused on the general mechanisms of inflammation, late they noticed that there were several miRNAs capable of regulating cytokines involved in the inflammatory response of the CD [78]. Since this discovery of miRNAs, several recent papers have revealed that miRNAs can also play a role in CD pathogenesis. It is well known that miRNAs are involved in the development of mature immune cells as well as controlling their functions, which suggest that these molecules may also be implicated in the development of inflammatory and autoimmune diseases [14]. For this reason, miRNAs can help improving the understanding of the CD pathophysiology as well as allowing new therapeutic targets and that they are also noninvasive biomarkers for the diagnosis of disease activity, severity, treatment response, and degeneration associated with IBD [75].

To date, several papers have focused investigations on the altered expression of miRNAs in CD and their important role as regulators and possible diagnostic biomarkers in CD [14–17]. The majority of studies in CD have been conducted in tissue and cellular cultures, and there are currently few reports on the quantitative assessment of circulating miRNA in IBD patients [79–82]. These works have identified peripheral blood miRNAs expression profiles in CD patients [79, 81] and have demonstrated their potential utility as noninvasive biomarkers [80].

The first study where miRNAs were directly examined in the mucosa of ulcerative colitis patients (the other major form of IBD) was performed by Wu et al. [83] in 2008. They examined miRNA expression in sigmoid colon biopsies from patients with ulcerative colitis (active and inactive), chronic active CD, irritable bowel syndrome, microscopic colitis, and healthy control subjects. They reported differential expression of miRNA in the mucosa of patients with active ulcerative colitis tissues compared with the rest of patients. This work demonstrated that miR-192 was predominantly expressed in colonic epithelial cells and was able to suppress expression of the macrophage inflammatory peptide-2-*α* (MIP-2*α*). In addition, they discovered that, in a colonic epithelial cell line, TNF-*α* induced the stimulation of MIP-2-*α* and blocked miR-192 expression.

Following publication of this study, other works have emerged aiming to identify all of the miRNAs dysregulated in CD to elucidate the expression patterns in the diverse CD subtypes and to identify the targets of the miRNAs involved in CD. In this way, Wu et al. have also identified different expression patterns among tissues from different intestinal regions and suggested that miRNAs are involved in different pathogenic mechanisms of IBD subtypes [84]. Several miRNAs could distinguish CD from ulcerative colitis (miR-19b, miR-106a, and miR-629). The authors suggested that miR-191 could be new noninvasive biomarkers to distinguish ulcerative colitis and CD [37, 85]. Previous studies have found that there are different miRNAs expression patterns between patients with active and inactive disease [14, 79, 82] and it has been demonstrated that the expression patterns differ between inflamed and noninflamed mucosa of CD patients [86, 87]. However, it seems that circulating miRNA profiles do not correlate with tissue miRNA profiles in active and inactive CD patients [82].

Intestinal fibrosis with stricture formation is a major complication in CD, which may require surgery. The traditional mechanisms underlying intestinal fibrosis are associated with the presence of chronic inflammation. However, it is also possible that novel mechanisms independent of persistent immune activation exist in the gut. Some publications have demonstrated the existence of miRNAs that would be associated with fibrotic processes including the family of miR-200 and miR-29 [88, 89]. Chen et al. demonstrated that specific miR-200b can improve fibrosis by induction of transforming growth factor beta 1 (TGF-*β*1) [88]. Moreover, the authors observed that the expression of miR-200b was increased significantly in the serum of the CD patients with fibrosis and that there are functional associations between miR-200 and key effectors of the epithelial-to-mesenchymal transition (EMT). EMT may promote intestinal fibrogenesis, which is probably inhibited by miR-200b [88].

Another study observed that the overexpression of miR-29b in CD fibroblasts led to a downregulation of collagen I and III transcripts and collagen III protein but did not alter MMP- (matrix metalloproteinase-) 3, MMP-12, and TIMP- (tissue inhibitor of metalloproteinase-) 1 production. TGF-*β*1 upregulated collagen I and III transcripts and collagen III protein as a consequence of the downregulation of miR-29b, and TGF-*β*1-induced collagen expression was reversed by exogenous overexpression of miR-29b. In addition, patients with structuring disease had lower serum levels of miR-29 than those without [89].

Another important consideration is the identification of possible biomarkers predictive of the therapeutic effect. In this sense, Fujioka et al. have identified two miRNAs, let-7d and let-7e, as possible therapeutic biomarkers in patients with CD, who were treated by anti-TNF drug (infliximab). These miRNAs showed to have a similar expression pattern according to the therapeutic effect of infliximab. The levels were significantly increased in the group of patients who achieved clinical remission by infliximab [90]. In addition, studies have demonstrated a significant role of let-7 miRNAs in the regulation of apoptosis through the inhibition of Fas and Bcl-xL.

## 4. Conclusions

We have reviewed here some recent points of research on oxidative stress biomarkers in blood and its correlations with Crohn's disease activity. We have discussed when and how it might be useful to analyze these events in CD patients, their possible biomarker capacity, and what implications they might have in the future management of the disease. In this sense, we have also indicated our experience in characterizing the ROS species generated in the peripheral blood mononuclear cells of CD patients (active and inactive) and the status of antioxidant enzyme activities (how they are expressed or modified) in both activity and remission phases of the disease.

The above highlights the fact that miRNA could be implicated in the pathogenesis of IBD. miRNA is expressed differentially in diverse circumstances and opens new opportunities to employ miRNA as an excellent biomarker for activity, diagnosis, severity, therapeutic response, and even degeneration associated with IBD. Researchers worldwide are interested in miRNAs as potential therapeutic targets and potential noninvasive tests for CD patients.
